# Explicit and indirect, latency-based measure of aggression in striking combat sports

**DOI:** 10.3389/fpsyg.2024.1451244

**Published:** 2024-08-13

**Authors:** Radu Predoiu, Andrzej Piotrowski, Elena Amelia Stan, Mihai Valentin Ciolacu, Andrei Bitang, Doina Croitoru, Germina Cosma

**Affiliations:** ^1^Department of Teacher Training, Faculty of Physical Education and Sports, National University of Physical Education and Sports, Bucharest, Romania; ^2^Institute of Psychology, University of Gdańsk, Gdańsk, Poland; ^3^Faculty of Physical Education, Sport, and Physiotherapy, Romanian–American University, Bucharest, Romania; ^4^Faculty of Psychology and Educational Sciences, University of Bucharest, Bucharest, Romania; ^5^Faculty of Physical Education and Sports, "Aurel Vlaicu" University of Arad, Arad, Romania; ^6^Department of Sports and Motor Performance, Faculty of Physical Education and Sports, National University of Physical Education and Sports, Bucharest, Romania; ^7^Faculty of Physical Education and Sports, University of Craiova, Craiova, Romania; ^8^InnovaSport Craiova Interdisciplinary Laboratory, INCESA, Craiova, Romania

**Keywords:** explicit aggression, martial arts, indirect aggression, IAT, sports performance

## Abstract

**Introduction:**

Aggression in sports is often perceived as a necessary trait for success, especially in martial arts. Aggression can be assessed both explicitly and implicitly, taking into account the dual processing model. The purpose of the research was to examine explicit and indirect, latency-based measure of aggression in competitive athletes practicing striking combat sports, according to gender and sports performance. At the same time, we verified whether aggression (implicit/unconscious and explicit) predicts sports performance in martial artists.

**Materials and methods:**

A total of 85 athletes practicing striking combat sports took part in the research. For implicit, latency-based measure of aggression, an Implicit Associations Test (IAT) was used, while explicit aggression was assessed with the Romanian adaptation of the Makarowski’s Aggression Questionnaire for martial arts athletes.

**Results:**

Data analysis revealed (using multivariate analysis of variance) that athletes from striking combat sports having international sports performances registered significantly higher D-scores (IAT, *p* = 0.014) and lower values for Go-ahead factor (*p* = 0.006), compared to athletes without outstanding results. Goodman and Kruskal tau association test was used to check the existing associations between athletes’ gender and martial arts athletes’ level of explicit and implicit aggression. In addition, binomial logistic regression procedures were performed, predicting martial artists’ likelihood to obtain higher sports results, based on explicit and indirect aggression.

**Conclusion:**

A stronger association between Aggression and Others (at implicit/unconscious level) and a moderate level (generally) for Go-ahead factor of explicit aggression are associated with an increased likelihood of sports performances in athletes. In addition, male martial arts athletes are more persistent despite obstacles, remaining more on the offensive (no gender-related association were found in terms of indirect/unconscious aggression, and for Foul Play and Assertiveness factors of explicit aggression). The study underlines the importance of addressing athletes’ subconscious level to promote more constructive behaviors in competitions.

## 1 Introduction

Considering the forms of direct confrontation (see Predoiu et al., 2022a; Patenteu et al., 2023a), combat sports and martial arts can be divided in striking combat sports (e.g., karate, taekwondo, boxing, kickboxing, and fencing—working with weapons in this case) and grappling combat sports (e.g., jiu-jitsu, judo, and wrestling). Striking combat sports (SCS) represent heuristic sport disciplines where athletes must be aggressive and make quick and appropriate decisions to win. On the relationship between martial arts and combat sports, Kalina (2000) mentioned “every combat sport is martial arts but not vice versa” (p. 18).

Martial arts can be defined as “systems that blend the physical components of combat with strategy, philosophy, tradition, or other features, thereby distinguishing them from pure physical reaction” (Green and Svinth, 2010, p. 19). Despite the aggressive nature of athletes to win, combat sports convey moral values, a key element during training being the ethical, social, and moral development of practitioners (Kostorz and Sas-Nowosielski, 2021). In combat sports, the aim is to achieve non-aggressive goals (winning) through aggressive behavior (Martinkova and Parry, 2016).

Aggression can be defined as a negative behavioral trait that can be reflected in harmful physical or mental behavior against others (Keeler, 2007). In this context, defined as the intention to physically or psychologically harm someone who is motivated to avoid such treatment, aggression can be either hostile or instrumental (Wann, 2005). If the aggressive behaviors of athletes are within the boundaries/rules of the game (and not hostile—see, also, Silva (1983) for hostile and instrumental aggression), leading to a positive competitive outcome, this type of aggression is applauded and socially appreciated (Cashmore, 2008; Patenteu et al., 2023b). In sport context, it is important to distinguish between the types of aggression (Rydzik, 2022). Instrumental aggression can be observed, generally, in sports activities. Its goal is to score a point or to stop a rival from gaining an advantage, while the competitor complies with the rules. In this instance, the sole prerequisite is the absence of any desire to cause harm to others, or the display of hostile/violent aggression; however, in the case of instrumental aggression, it still has an intentional and calculated character (Krishnaveni and Shahin, 2018).

Research suggests that a combination of individual factors, such as personality traits (e.g., high levels of competitiveness or low impulse control), and situational factors, such as the competitive nature of the sport or the high stakes involved, can contribute to the occurrence of aggression in sports (Russell, 2008; Newman et al., 2021). Hernandez and Anderson (2015) investigated aggression, in martial arts, within the framework of the general aggression model (GAM) theory. In accordance with GAM (Anderson and Bushman, 2018), the aggressive behavior is influenced by one’s decisions, arousal, by the biological factors together with the persistent environmental characteristics, by the existing cognitions and feelings, changing the knowledge structures of the individual. The significant role of the social knowledge structures in aggressive behavior is well known (Dodge, 1980). In sports, the persistent use of aggressive behaviors along with the trainer’s encouraging reinforcement leads to an increase in aggression (Syrmpas and Bekiari, 2018). Frequently, athletes are encouraged by the coach to play a tough game, especially if it brings success. According to Petrovska et al. (2021), training in aggressive actions due to the specifics of activity and constant conflict of situations leads, as a rule, to an increase in the level of aggression of an athlete. In these conditions, it is very important that the athlete possess those qualities that would allow him/her to govern and control aggression. The social and cultural influences, the aggressive role models, shape, therefore, aggressive behavior in sports (Marwat et al., 2022).

### 1.1 Explicit and indirect measure of aggression

Aggression can be assessed both explicitly and implicitly (Gollwitzer et al., 2007) taking into account the dual processing model (Gawronski and Bodenhausen, 2006), existing two ways of processing information (Strack and Deutsch, 2004). Automatic (implicit) processing occurs in the absence of conscious control, individual’s behavior being the result of the activation from memory of a set of associations (De Houwer et al., 2009). Explicit aggression involves overt, easily observed, direct manifestations, for example, verbal manifestations/threats and physical violence (Neuman and Baron, 1998). Implicit or indirect aggression is subtle, being less visible and obvious, can be triggered by situational characteristics (Todorov and Bargh, 2002), and being conceptualized as an automatically activated self-attitude (Uhlmann and Swanson, 2004; Gollwitzer et al., 2007). Implicit measurements explore a combination of traits and states, which are subjected to variation generated by situation-specific circumstances (Dasgupta and Greenwald, 2001), specialized literature emphasizing that aggression research might benefit from measuring (automatic) reactions (Blümke and Zumbach, 2007).

Human behavior is guided, therefore, by implicit and explicit processing (Gawronski and Bodenhausen, 2006), being essential to consider the role of both indirect, latency-based, and explicit measure of aggression. Both automated and conscious/deliberate ways of processing information contribute differentially and define aggressive behavior (Richetin and Richardson, 2008). Explicit aggression can be assessed through questionnaires, while indirect aggression can be measured with indirect measurement tools, for example, implicit association test (IAT).

IATs to measure implicit attitudes toward sport in elite athletes (Gerber et al., 2019), implicit exercise importance (Forrest et al., 2016), or beliefs about sport ability in basketball and swimming (Mascret et al., 2016) have been used. However, to the best of our knowledge, very few articles explored IAT ability to predict sports performance (aggression being investigated), compared to explicit assessments, for example, Teubel et al. (2011) in basketball players and Predoiu et al. (2022b) in martial arts coaches. We mention, also, that IATs to measure implicit aggression have been used in previous research, with school children (Blümke and Zumbach, 2007) and adolescents (Gollwitzer et al., 2007), but unrelated to sports performance.

## 2 The current study

The aim of the research was to examine explicit and implicit aggression in competitive athletes practicing striking combat sports, according to gender and sports performance. At the same time, we wanted to verify whether aggression (unconscious/automatic aggression and also, explicit) predicts sports performance in martial artists.

### 2.1 Objectives

The objectives were as follows:

- Knowing the explicit and implicit aggression in martial artists from striking combat sports;
- Identifying the differences between martial artists, in terms of explicit and automatic aggression, taking into consideration athletes’ sports performances;
- Establishing gender-related associations, in the case of martial artists, regarding direct and indirect aggression;
- Identifying predictors of sports performance, in the case of martial artists, starting from athletes’ implicit and explicit aggression.

*Hypotheses*

The hypotheses were as follows:

*H*_1_: Investigation of explicit and indirect, latency-based measure of aggression reveals significant differences between martial arts athletes according to sports performances.

*H*_2_: There are significant associations between athletes’ gender and martial arts athletes’ level of explicit and implicit aggression.

*H*_3_: The results for implicit/automatic aggression represent a better predictor of sports performance, among martial artists, than the results obtained for explicit measure of aggression.

## 3 Materials and methods

### 3.1 Participants

A total of 85 athletes practicing striking combat sports, affiliated to different sports clubs in Romania, took part in the research, 62 male and 23 female athletes, aged 18–28 years (*M*_age_ = 22.1, *SD* = 3.06). Inclusion criteria were minimum 18 years old (seniors) and minimum 2 years of competitive experience. Athletes have been practicing martial arts for an average of 7.41 years, *SD* = 3.28, and have between 2 and 9 years of competitive experience (*M* = 4.68, *SD* = 2.28, in the entire sample). Martial artists examined are systematically involved in training and competitions. The distribution of martial arts athletes according to sport discipline can be found below:

- karate: 28 athletes (32.9%)—19 male (M) and nine female athletes (F);
- kickboxing: 15 (17.6%)—13 M and 2 F;
- boxing: 14 (16.5%)—11 M and 3 F
- taekwondo: 14 (16.5%)—11 M and 3 F;
- fencing (Olympic combat sport, see Bagińska et al., 2022): 14 (16.5%)—8 M and 6 F.

According to division of the combat sports under forms of the direct confrontation (Kalina, 2000): hits (karate, kickboxing, boxing, taekwondo, etc.); workings of weapons (fencing); throws and grips of immobilization of opponent’s body (e.g., Brazilian jiu-jitsu, judo, and freestyle wrestling). In the current study, we analyze hits and workings of weapons as conventional “striking combat sports,” the same as in previous research (Predoiu et al., 2022a).

In terms of sports performances, athletes registered the following:

- International level performances (top ranks at World and/or European competitions): 28 athletes (32.9%) of which eight female athletes;
- National level results (top ranks at national competitions): 32 (37.6%) of which eight female athletes;
- Regional/local level sports results (at county level): 25 (29.4%) of which seven female athletes.

Athletes having international or national level performances were classified as elite/experts (according to the athletes’ highest standard of performance—Swann et al., 2015), while a second group obtained regional or local level results.

The snowball sampling technique was used to identify and examine senior athletes practicing striking combat sports, boys and girls with minimum 2 years of competitive experience and various sports performances (international, national, and regional/local results).

### 3.2 Measures

For indirect, latency-based measure of aggression, an Implicit Associations Test (IAT) was created, using the classic 7-block version (Greenwald et al., 1998) and the 20 + 40 trials subdivision (Schnabel et al., 2008). Table 1 emphasizes the 7-block functioning, including the number of trials in each block and items assigned to the right-and left-key response. The IAT lasts 16 min.

**Table 1:** Sequence of blocks in the IAT.

Block	No. of trials	Function	Items assigned to left-key response	Items assigned to right-key response
1	20	Practice	Aggression	Non-aggression
2	20	Practice	Self	Others
3	20	Test	Aggression + Self	Non-aggression + Others
4	40	Test	Aggression + Self	Non-aggression + Others
5	20	Practice	Others	Self
6	20	Test	Aggression + Others	Non-aggression + Self
7	40	Test	Aggression + Others	Non-aggression + Self

According to Greenwald et al. (1998), the Implicit Association Test (IAT) is an online metric of response time that assesses unconscious/implicit associations between particular concepts. Numerous specialists have used IAT to investigate different forms of implicit social cognition (Greenwald and Banaji, 1995). In an IAT, categories (e.g., “Aggression,” “Non-Aggression,” “good,” and “bad”) appear on the right versus left side of the screen, the participants assigning different stimuli (e.g., “fair play,” “insult,” “threat,” and “patience”) to the correct category by pressing the appropriate button on the keyboard (specified in the test instructions). IAT is a computerized dual-categorization task (a reaction-time-based classification task), the participants having to respond as quickly and accurately as possible (Sukhera et al., 2019). Richetin et al. (2010) suggested that IATs might reflect aggressive tendencies and intentions to harm in very indirect forms of aggression. Using an IAT, Grumm et al. (2011) measured “the association between the concept of self and the attribute aggressive by contrasting reaction times from two different response tasks.” The final score (D-score) reflects the intensity of implicit associations between categories, which could be low, moderate, strong, or no association at all.

It seems that the IAT is less susceptible to faking than questionnaires (an explicit measure)—Steffens (2004), insensitive to procedural variation (Nosek et al., 2005), and demonstrated high test–retest reliability and good internal consistency (Greenwald and Nosek, 2001; Nosek et al., 2007). Researchers demonstrated the aggressiveness-IAT’s ability to predict aggressive behavior beyond standard self-reported measures (Greenwald et al., 2009).

The IAT is a popular means of examining “hidden” biases, multiple versions of the Implicit Associations Test being created, investigating biases relating to race, age, or illness category, but all operating on the same principles (Sukhera et al., 2019). It seems that the predictive validity of the Implicit Associations Test can be affected by individual moderators (Nosek, 2005; Friese et al., 2008a), or contextual ones (Greenwald et al., 2009). In addition, it was found that IAT predicts better impulsive behaviors (Friese et al., 2008b).

In the present research, the following categories were used: Non-aggression, Aggression, Others, and Self. In IATs is common to use the object dimension Self-Others (Banse et al., 2015), the words specific to these categories being translated from previous studies (Greenwald and Farnham, 2000; Banse et al., 2015). In the current IAT, all words were in Romanian language.

The attributes are as follows: I, mine, my, me, self (for Self category); theirs, they, them, their, other (for Others category); threat, swear, hit, beat, insult (for Aggression category); fair play, discipline, respect, fairness, encouragement (for Non-aggression category). The IAT in the present study was used, also, in previous research with martial arts coaches (Predoiu et al., 2022b). For the detailed procedure validating the choice of appropriate attributes in the case of Aggression and Non-aggression categories, see Predoiu et al. (2022b).

To calculate the score on IAT, the improved D-scoring algorithm with error penalty 600 ms, labeled as D_4_ (see Greenwald et al., 2003), was used. According to D_4_, in the case of an error, it was replaced with mean(C) + 600 ms, where mean(C) is the block mean of correct-response latencies. A negative D-score highlights a stronger association between Aggression and Others, and a positive D-score highlights a stronger link between Aggression and Self.

In the case of D-score, specialized literature presents standard cutoffs (Chassot et al., 2015; Lee, 2017): 0.35 means a moderate link, 0.15 a slight/weak association, and 0.65 a strong association. However, being aware of the findings of Blanton et al. (2015), which discussed about the arbitrary character for the categorizations of D-scores, in the present study we will consider a weaker or a stronger association between Aggression and Self, or between Aggression and Others, avoiding being categorical in interpreting the D-score (as highlighting a moderate, weak or strong association). As Klein (2020) mentioned, even if the IAT and the D-scores spark controversy, the individual D-scores can reveal essential patterns at the group level.

The results of trials within the same test (*n* = 85) were used to calculate internal consistency. Considering IAT, Cronbach’s alpha coefficient (*α*) value was 0.67 in the current research.

Explicit aggression was assessed with the Romanian adaptation of the Makarowski’s Aggression Questionnaire for martial arts athletes (Makarowski et al., 2021). The questionnaire consists of 15 items and evaluates 3D: Go-ahead, Assertiveness, and Foul Play. There are five items for each scale (no reverse-scored items). Athletes answered on a 5-point Likert scale, from 1—“Definitely not,” to 5—“Definitely yes.”

Considering “Go-ahead” factor, the athlete attacks, is courageous, breaks obstacles (e.g., “There is no argument that would turn me away from reaching my goal”). Assertiveness, as a personality characteristic, assumes that athletes are acting within the boundaries of the game, which can lead to success (Bredemeier, 1994), expressing themselves verbally or behaviorally in a constructive way, firmly but without offending others (opponents, peers, referees)—for example, “I’m not afraid to speak up to my supervisor or coach, if I know that he/she is wrong.” Regarding “Foul play” factor of aggression, the athlete has no scruples, achieving the goal (winning) is what matters, regardless of the means, and, therefore, is willing to act in an unethical manner (outside the rules of the game)—for example, “I think that *anything goes* rule is appropriate to achieve the victory.”

The internal consistency/reliability for the three factors of aggression investigated, in the present research, measured with Cronbach’s alpha coefficient (*α*) was 0.74 (Go-Ahead), 0.76 (Assertiveness), and 0.73 (Foul Play), respectively.

### 3.3 Procedure

In the early stages of the research, 94 eligible athletes (in terms of age and competitive experience) practicing striking combat sports were examined, but 85 remained for future analysis. Three martial arts athletes were removed from the study because they exceeded critical error rates of 35% in one of the combined blocks in the IAT, the same as in Banse et al. (2015). In addition, six athletes were excluded from the study having |D| > 0.65 (see Klein, 2020), “in order for the confidence intervals (in the case of D-scores) to span below the 0.65 cutoff, meaning a slight or moderate bias (not a strong bias)” (Predoiu et al., 2022b).

The IAT and the questionnaire use to evaluate explicit aggression were carried out in the period of 2023–2024, in the presence of the experimenter. The study was conducted in Romania. Athletes from striking combat sports completed the questionnaire (including socio-demographic data and regarding the highest sports performance registered) via Google forms (Google LLC, Mountain View, CA, United States). The IAT was made on a computer, with the help of the PsyToolkit platform (Stoet, 2010; Stoet, 2017). Athletes completed the test between 2 and 7 p.m. Similar, computers were used in the research, the screen resolution being the one recommended by the computer (1920 × 1,080 pixels). The research is cross-sectional (Predoiu, 2020) and is based on *ex post facto* design (Thomas and Nelson, 2001).

The correlations between D-score (IAT) and the scores obtained in the case of direct measure of aggression (the values for the three factors of explicit aggression) were very low (Pearson’s *r* = 0.009, *p* = 0.935 – IAT/Go-Ahead; *r* = 0.08, *p* = 0.465 – IAT/Foul Play; *r* = 0.13, *p* = 0.248 – IAT/Assertiveness). This is in line with the specialized literature on associations between direct and indirect measures (see, e.g., the results of the meta-analysis by Hofmann et al., 2005). As Banse et al. (2015) asserted, also, low correlations with self-reported aggressiveness reflect the usefulness of indirect measure.

### 3.4 Statistical analysis

IBM SPSS Statistics Version 27.0 (Armonk, NY, IBM Corp) and Jamovi were used for the statistical analysis. In the case of MANOVA, Scheffe *post-hoc* test was reported due to Levene’s test results (equality of variance, *p* > 0.05)—Popa (2010). *t*-test for independent samples was also used. Variables were normally distributed, with Skewness and Kurtosis coefficients (in absolute value) being <2 (George and Mallery, 2010). Following the recommendations of Argyrous (2005), Goodman and Kruskal tau association (an asymmetric test) was performed, at least one variable being categorical. Cramer’s V coefficient (the effect size index) for 2 × 3 tables was interpreted: 0.10—weak association, 0.30—moderate, 0.50—strong association (Nyberg et al., 2023). Not least, analysis of the results involved using binomial logistic regressions, Nagelkerke *R*^2^ (effect size) being interpreted as follows: 0.35 large effect size, 0.15 medium, 0.2 small effect size (Cohen, 1992).

## 4 Results

Stem-and-leaf and box-plot analysis revealed that no outliers were identified (preliminary data analysis). In the case of the present study, athletes automatically associated Aggression with Others, obtaining negative D-scores, the same as in previous research when martial arts coaches were examined, maybe due “to the words designated as representative for aggression in sports […] (threat, beat, hit, swear and insult),” which are, generally, closer to hostile aggression (Predoiu et al., 2022b).

The results registered by martial artists from striking combat sports for the dependent variables examined (at descriptive level), according to sports performances, are presented in Table 2.

**Table 2:** Descriptive statistics—implicit and explicit aggression (*n* = 28, international results, *n* = 32, national performances, and *n* = 25, regional/local sports results).

D-score (indirect aggression)	International results	Mean	0.41
		SD	0.13
	National performances	Mean	0.32
		SD	0.17
	Regional/local results	Mean	0.28
		SD	0.16
Go-ahead	International results	Mean	18.64
		SD	2.68
	National performances	Mean	18.47
		SD	3.27
	Regional/local results	Mean	21.16
		SD	2.09
Foul play	International results	Mean	8.50
		SD	2.95
	National performances	Mean	7.97
		SD	3.71
	Regional/local results	Mean	9.92
		SD	3.55
Assertiveness	International results	Mean	16.93
		SD	3.66
	National performances	Mean	16.75
		SD	5.52
	Regional/local results	Mean	17.16
		SD	3.07

Using multivariate analysis of variance, the differences between martial artists (in the disciplines of Striking) were verified, in terms of explicit and indirect, latency-based measure of aggression. Type I procedure (for group inequality) was selected for MANOVA. The *p*-value in the case of the Box M test is 0.012, the Wilks’ Lambda test values being reported: Wilks’ Lambda = 0.739, *F*(8, 158) = 3.228, *p* = 0.002, eta^2^ = 0.14, observed power = 0.966. The Test of Between-Subjects Effects emphasizes that sports performance significantly influences the values for D-score (*F* = 4.837, *p* = 0.010, Partial Eta Squared = 0.10) and Go-ahead (*F* = 7.831, *p* = 0.001, Partial Eta Squared = 0.16). The homogeneity of variances condition was met, results for Levene’s test: *F* = 1.302, *p* = 0.278 for D-scores, respectively, *F* = 2.582, *p* = 0.082 in the case of the Go-ahead factor of explicit aggression. Table 3 presents only the significant differences observed (Scheffe *post-hoc* test was interpreted).

**Table 3:** *Post-hoc* Scheffe test—single-factor MANOVA.

Dependent variable		(I) Sports performance	(J) Sports performance	Mean difference (I–J)	*p*	95% confidence interval	
						LB	UB
D-score	Scheffe *post-hoc* test	I	N	0.0914	0.089	−0.0107	0.1936
			L/R	0.1305^*^	0.014	0.0218	0.2391
		N	I	−0.0914	0.089	−0.1936	0.0107
			L/R	0.0390	0.654	−0.0664	0.1444
		L/R	I	−0.1305^*^	0.014	−0.2391	−0.0218
			N	−0.0390	0.654	−0.1444	0.0664
Go-ahead		I	N	0.17	0.971	−1.62	1.96
			L/R	−2.52^**^	0.006	−4.42	−0.61
		N	I	−0.17	0.971	−1.96	1.62
			L/R	−2.69^**^	0.002	−4.54	−0.84
		L/R	I	2.52^**^	0.006	0.61	4.42
			N	2.69^**^	0.002	0.84	4.54

I, international sports performances; N, national results; L/R, local/regional performances; LB, lower bound; UB, upper bound, ^*^p < 0.05; ^**^p < 0.01.

In the case of implicit aggression, and for Go-ahead factor, significant differences were observed between athletes taking into consideration sports performances. Martial arts athletes from striking combat sports having international performances registered significantly higher D-scores (*p* = 0.014, *M*_INTERNATIONAL_ = 0.41, *SD* = 0.13) and significantly lower values for Go-ahead factor (*p* = 0.006, *M*_INTERNATIONAL_ = 18.64, *SD* = 2.68), compared to athletes without outstanding results: *M*_LOCAL/REGIONAL_ = 0.28, *SD* = 0.16 in the case of IAT, respectively *M*_LOCAL/REGIONAL_ = 21.16, *SD* = 2.09 for Go-ahead (Table 3). It is worth mentioning that the results for Go-ahead (athletes having international sports results) are, generally, moderate, according to the norms (Makarowski et al., 2021). In addition, athletes having national performances obtained significantly lower scores for Go-ahead factor (*p* = 0.002), compared to martial artists having local or regional sports results.

Next, the existing associations between athletes’ gender and martial arts athletes’ level of explicit and implicit aggression were verified. Goodman and Kruskal tau association test was used (at least one variable being categorical, the results for aggression—explicit and indirect representing the dependent variables). Table 4 includes only the significant association identified.

**Table 4:** Directional measures*—*Goodman and Kruskal tau association.

		Value	Asymp. std. error	Approx. sig.
Goodman and Kruskal tau	Gender	0.094	0.067	0.019
	Go-ahead dependent	0.050	0.036	0.015
Crosstabulation gender and go-ahead factor of aggression				
	Go-ahead			Total
	Low	Average	High	
Male martial arts athletes	15	30	17	62
Female martial arts athletes	13	6	4	23
Total	28	36	21	85

Out of the 62 male martial arts athletes, 30 athletes (48.3%) obtained average scores in the case of the Go-ahead factor of aggression, while 17 (or 27.4%) registered high values. Male athletes tend to obtain average and high scores for the “perseverance in reaching the goal despite numerous obstacles […]. They are expansive and dynamic. Athletes high in go-ahead are bold, remain on the offensive, and they do not hesitate” (Makarowski et al., 2021). In the case of the 23 female martial artists from striking combat sports, 13 athletes (56.5%) obtained low scores (Go-ahead factor), while six (26%) registered average values.

In the Directional measures table (Table 4) one can observe the Goodman and Kruskal tau association coefficient (0.050) and the adjacent threshold of significance (*p* = 0.015). A significant association can be highlighted between athletes’ gender and the results for Go-ahead. Cramer’s V coefficient is 0.306, emphasizing a moderate link between variables. In the case of implicit/automatic aggression and for the other factors of explicit aggression investigated (Foul Play and Assertiveness), no gender-related association was found.

In the next phase, knowing that indirect, latency-based measure of aggression and Go-ahead are specific to martial arts athletes having international sports results (at World and European level), the extent to which the two dimensions predict sports performances was investigated (*n* = 53). Two separate binomial logistic regressions were performed (Tables 5–8).

**Table 5:** Binomial logistic regressions analysis—IAT (D-scores).

	Implicit aggression
Omnibus tests of model coefficients (Chi-square and *p*)	9.141 (0.002)
Overall percentage (predicted—percentage correct)	66% (accuracy)
Regional/local performances (predicted)	64% (specificity)
International performances (predicted)	67.9% (sensitivity)
Nagelkerke *R*^2^	0.212
Hosmer and Lemeshow test: Chi-square (*p*-value)	6.294 (0.614)

**Table 6:** Binomial logistic regressions analysis—variables in the equation (IAT).

	B	S.E.	Wald	df	*p*	Exp(B)/odds ratio	95% CI for Exp(B)	
							Lower	Upper
D-score constant	5.70	2.088	7.450	1	0.006	298.7	4.987	17895.3
	−1.90	0.801	5.625	1	0.018	0.150		

**Table 7:** Binomial logistic regressions analysis—Go-ahead (explicit aggression).

	Explicit aggression
Omnibus tests of model coefficients (Chi-square and *p*)	12.504 (0.000)
Overall percentage (Predicted—Percentage correct)	69.8% (accuracy)
Regional/local performances (predicted)	56% (specificity)
International performances (predicted)	82.1% (sensitivity)
Nagelkerke *R*^2^	0.281
Hosmer and Lemeshow test: Chi-square (*p*-value)	15.172 (0.084)

**Table 8:** Binomial logistic regressions analysis—variables in the equation (go-ahead factor).

	B	S.E.	Wald	df	*p*	Exp(B)/odds ratio	95% CI for Exp(B)	
							Lower	Upper
Go-ahead constant	−0.410	0.131	9.779	1	0.002	0.664	0.513	0.858
	8.260	2.625	9.903	1	0.002	3866.4		

The models are significant (*p* < 0.05, Omnibus tests of model coefficients—Tables 5, 7). The *p* > 0.05 in the case of the Hosmer and Lemeshow goodness of fit test, meaning that the models are not a poor fit. The models correctly classified 66% (IAT) and 69.8% (Go-ahead) of cases.

Nagelkerke *R*^2^ highlights a moderate to strong relation (*I*^2^ = 0.21 – IAT, *R*^2^ = 0.28—Go-ahead) between each dimension (implicit and explicit aggression) and sports performances.

In the case of martial artists from striking combat sports, both results—for implicit/indirect and explicit aggression (Go-ahead factor), represent important predictors of sports performance, representing valuable resources for sports psychologists, coaches, and athletes. A moderate level (generally) for Go-ahead and a stronger association between Aggression and Others (at implicit/unconscious level) are associated with an increased likelihood of international sports results in martial arts athletes.

## 5 Discussion

Sports, as a competitive activity, often involves a certain level of physicality and intensity, which can lead to the emergence of aggression within the sporting context (Krishnaveni and Shahin, 2018). Aggression in sport is determined by the most particular elements of this domain: high conflict scenarios, the thrill of certain sports, and the intense nature of competition (Bekiari et al., 2015). Aggressive behavior, in some sports (martial arts and sports games) is one of the instrumental means of achieving the main goal (Petrovska et al., 2021), instrumental aggression serving as a competitive advantage, but aggression in athletes combined with the desire to win can cause, also, violations of the rules of competition and departure from the rules of fair competition (Graczyk et al., 2010; Kostorz and Sas-Nowosielski, 2021).

In a first phase, the differences between martial artists were explored, taking into consideration sports performances. Significant differences were observed in the case of implicit/unconscious aggression, and for Go-ahead factor of explicit aggression, between athletes practicing striking combat sports. Martial arts athletes having international and national performances registered significantly lower values for Go-ahead factor, compared to athletes without outstanding sports results. It is worth mentioning that the results for Go-ahead (athletes having international or national performances) are, generally, moderate (according to the norms). These results can be explained through the Yerkes-Dodson law, a moderate level of arousal (Go-ahead factor in this case) enhancing performance in complex tasks (Chaby et al., 2015), and sport is characterized by stress (Mellalieu et al., 2009) and complex tasks. Martial artists who attack, persevere in achieving their objectives despite obstacles, and remain on the offensive (at a moderate level, generally) registered higher sports results in competitions. Previous research with Romanian martial arts athletes having performances at international and national level (Makarowski et al., 2021) underlined, also, an average level (generally) for Go-ahead dimension. Zivin et al. (2001) asserted that more experienced athletes (especially in martial arts) channels aggression in a more constructive way, controlling it better, while novices manifest higher levels of explicit aggression due to anxiety related to their new activity (Smith and Smoll, 1990). In addition, experienced athletes tend to perceive aggression more as a strategic component, while novices may show a lower level of self-control and higher levels of uncontrolled aggressive impulses (Jones et al., 2002). However, Rui and Cruz (2017) found no correlation between athletes’ explicit aggression and sports performances, the results being inconclusive and contradictory. The specific culture of the sport practiced can influence aggression in athletes. A training environment that emphasizes self-control, respect, and discipline (as in martial arts) may reduce aggression (Twemlow et al., 2008). In fact, researchers underlined the importance of practicing combat sports and martial arts in reducing aggression (Daniels and Thornton, 1992; Steyn and Roux, 2009; Vertonghen and Theeboom, 2010), emphasizing, in the same time, a lack of studies considering the aggressive behavior of martial artists (Chen et al., 2019). In addition, incorporating internal techniques (breathing and self-control) into martial arts training can lessen the impact of this training on aggressive impulses and behavior (Hernandez and Anderson, 2015). However, every athlete has his/her own facilitative level of arousal and aggression (sports performance being idiosyncratic). Further studies need to shed more light on the level of different factors of aggression that facilitates sports results in a given setting.

Athletes having international performances obtained significantly higher D-scores (IAT), compared to martial artists having local or regional sports results, associating at a higher level Aggression with Others, at an unconscious/automatic level. This is in line with martial arts’ coaches level of implicit aggression (martial arts coaches are, generally, former martial arts practitioners)—martial arts coaches having international sports results “automatically associated aggression with others at a higher level than novice coaches did” (Predoiu et al., 2022b). We argue that athletes having international sports performances (at World and/or European level), automatically associating Aggression (unconscious aggression) with Others at a stronger level (than athletes without outstanding results), manage to generate an emotional tension during trainings, closer to competitions, having embedded in their deep structures of the psychic system the information that sports performance is very difficult to be achieved, and all these in a stress-generating environment that sport entails (Gilbert et al., 2007). Stressors, in sport, often impact the way athletes feel, think, and behave in athletic field (Fraser-Thomas and Côté, 2009; Rice et al., 2016). Taking into consideration the General Aggression Model (GAM) theory (Anderson and Bushman, 2018), the knowledge structures of an athlete can be changed by the existing feelings and cognitions, by the persistent environmental/competition characteristics.

Furthermore, the existing associations between martial arts athletes’ gender and athletes’ level of explicit and implicit aggression were verified. A significant association was highlighted between athletes’ gender and the results for Go-ahead factor, the effect size index emphasizing a moderate link between variables. In the case of male martial arts athletes, most of them (48.3%) registered average scores (the Go-ahead factor of explicit aggression), while 27.4% obtained high values. Most of the female athletes from striking combat sports (56.5%) obtained low scores, while 26% registered average values. Coulomb-Cabagno and Rascle (2006) emphasized that regardless of the level of competition, or the type of practiced sport, female athletes display lower levels of explicit aggression than male athletes. In terms of implicit/indirect aggression, and for the other factors of explicit aggression investigated (Foul Play and Assertiveness), no gender-related association was found in martial artists.

Not least, knowing that indirect, latency-based measure of aggression and Go-ahead factor are specific to martial arts athletes having international sports results (at World and/or European level), the extent to which the two dimensions predict sports performance was examined. We can argue that in the case of martial artists from striking combat sports, both results (for implicit/indirect and explicit aggression—Go-ahead factor) represent important predictors of sports performance. The two dimensions represent valuable resources for specialists in the field on sports science and psychology, working with athletes. A moderate level (generally) for Go-ahead and a stronger association between Aggression and Others (at implicit/unconscious level) are associated with an increased likelihood of sports performances in martial arts athletes. Predoiu et al. (2022b) highlighted that “implicit aggression is a better predictor of sports performance than explicit (verbal) aggression,” in coaches. However: (a) both models—for explicit and indirect measure of aggression, were significant (the differences being small, in favor of implicit aggression); (b) Go-ahead factor did not predict sports performances, but verbal aggression did (in the case of martial arts coaches). Indirect measures (an Aggressiveness IAT was used) were found, also, to be a better predictor of sports performance than direct measures (Teubel et al., 2011), the study exploring semi-professional male basketball players.

The results address gaps in the literature considering the role of indirect/unconscious measure of aggression and its connection with sports performance. The development and application of the IAT represent an essential advance in understanding the psychological dimensions of athletes, such as aggression. As Blümke and Zumbach (2007) mentioned, “although there are still many methodological problems to overcome, implicit measures have already added to our knowledge of preactivation of emotional and cognitive content in social encounters,” with an increasing body of evidence for the predictive validity of IATs (see Greenwald et al., 2009, for an overview).

## 6 Limitations and directions for future research

The study is not without limits. For example, each striking combat sport discipline can be separately explored; therefore, larger samples should be examined in future and in a different setting (related to country and athletes’ age). In addition, each weight class in boxing, kickboxing, or taekwondo can be separately addressed. The results could be different if athletes from grappling combat sports will be investigated (e.g., jiu-jitsu, judo, and freestyle wrestling), or athletes practicing mixed martial arts (MMA) which combines techniques from both fighting styles—striking and grappling. Aspects such as athletes’ income, level of education, body mass index, or history of injuries can be, also, considered as limits of the current research. Regarding the injuries suffered by athletes, researchers found that athletes with a history of concussion were more impulsive (Goswami et al., 2016) and more physically aggressive (Gallant et al., 2018). Moreover, the phenomenon of rapid weight loss, commonly employed by combat sports athletes, should be addressed in future research. This phenomenon can lead to mood perturbations and perhaps enhance levels of aggression in athletes—for a systematic review considering the effects of rapid weight loss (RWL) on athletes’ mood states, and the psychological ramifications of RWL, see Lakicevic et al. (2024).

With respect to explicit aggression, martial arts athletes took part in the study via a Google questionnaire; in these conditions, a limitation of the research can be considered the relatively small sample size. However, the athletes completed, also, the IAT to assess implicit aggression in the presence of the experimenter. Further research should examine other dimensions of explicit aggression, such as physical aggression, anger, verbal aggression, or hostility (see Buss and Perry, 1992) and their role in predicting sports performance, in martial arts being found that higher levels of anger facilitate athletes’ performance (Terry and Slade, 1995; Wargo et al., 2007).

In addition, the results regarding the IAT (D-scores) could be different if other words were chosen as being representative for Aggression and Non-aggression, respectively, in the initial phases of the research, for example, warning (not threat), dispossession, or shout (not insults or swear). However, see Predoiu et al. (2022b) for the detailed procedure of stimuli selection in the current IAT, the selected words being in line with the opinion of sports specialists on what aggression and non-aggression in sport means. Not least, the conclusions could be different if various methods of measuring implicit/indirect aggression would be used (e.g., Conditional Reasoning Test or Picture Story Exercise).

Automatized assessments of individual’s cognitive processing in sports advance knowledge (Strenge et al., 2020), the study of indirect, latency-based measure of aggression (and not only), in athletes and coaches, representing a goal to be achieved in future studies.

## 7 Conclusion

In summary, a moderate level (generally) for Go-ahead factor of explicit aggression and a stronger association between aggression and others (at implicit/unconscious level) are associated with an increased likelihood of sports performances in athletes from striking combat sports. In addition, a significant and moderate association was highlighted between athletes’ gender and the results for Go-ahead factor. Male martial arts athletes are more expansive and dynamic, more persistent despite numerous obstacles, remaining more on the offensive. In terms of indirect/unconscious aggression, and for the other factors of explicit aggression investigated (foul play and assertiveness), no gender-related association was found.

The IAT offers a valuable window into understanding the complexities of aggression in sports. The differences between implicit/automatic and explicit/direct evaluations of aggression underscore the importance of addressing athletes’ subconscious level to promote more constructive behaviors in training, competitions, and everyday life.

## Data Availability

The raw data supporting the conclusions of this article will be made available by the authors, without undue reservation.
